# A New Route to Tune the Electrical Properties of Graphene Oxide: A Simultaneous, One-Step N-Doping and Reduction as a Tool for Its Structural Transformation

**DOI:** 10.3390/molecules30173579

**Published:** 2025-09-01

**Authors:** Andjela Stefanović, Muhammad Yasir, Gerard Tobías-Rossell, Stefania Sandoval Rojano, Dušan Sredojević, Dejan Kepić, Duška Kleut, Warda Saeed, Miloš Milović, Danica Bajuk-Bogdanović, Svetlana Jovanović

**Affiliations:** 1 Vinča Institute of Nuclear Sciences-National Institute of the Republic of Serbia, University of Belgrade, P.O. Box 522, 11000 Belgrade, Serbia; 2 Carl von Ossietzky Universität Oldenburg, 26129 Oldenburg, Germany; 3 Institut de Ciencia de Materials de Barcelona (ICMAB-CSIC), Campus de la UAB, 08193 Bellaterra, Barcelona, Spain; gerard.tobias@icmab.es (G.T.-R.); ssandoval@icmab.es (S.S.R.); 4 University of Belgrade, Faculty of Physical Chemistry, Studentski trg 12-16, 11158 Belgrade, Serbia; danabb@ffh.bg.ac.rs

**Keywords:** graphene, graphene oxide, N-doping, electrochemical exfoliation, density functional theory, electro-magnetic shielding

## Abstract

The presence of secondary electromagnetic waves (EMWs) results in EMW pollution and a large need for EMW-shielding materials. Therefore, new, lightweight, flexible, chemically resistant, and durable EMW shielding materials are demanded, while graphene and its derivatives meet the above-mentioned requirements. Among graphene derivatives, N-doped graphene exhibits promising electrical properties for shielding applications, although achieving sufficient N-incorporation in the graphene sheets remains a challenge. Herein, we produced graphene oxide using the modified Hummers’ method (GO) and the electrochemical exfoliation of highly ordered pyrolytic graphite. These two GO samples were thermally treated at 500 °C and 800 °C under a pure NH_3_ gas for 1 h. UV-Vis, infrared, and Raman spectroscopies and X-ray diffraction, elemental, and thermogravimetric analyses were used to investigate the structural properties of modified GO. One of the highest levels of N-doping of GO was measured (11.25 ± 0.08 at%). The modification under a NH_3_ atmosphere leads to simultaneous N-doping and reduction of graphene, resulting in the formation of electrically conductive and EMW shielding materials. Density functional theory (DFT) revealed the effect of heteroatoms on the energy band gap of GO. The cluster corresponding to N-doped rGO had a reduced bandgap of 0.77 eV.

## 1. Introduction

Electromagnetic waves (EMWs) have become widespread owing to the extensive use of electronic devices, such as mobile phones, wireless communication devices, artificial satellites, and radars [1]. As a result of electromagnetic wave pollution, various electronic and measuring equipment malfunctioned, and significant concerns were raised about its effects on public health [2]. The primary strategy for controlling EMW exposure is the use of electromagnetic interference (EMI) shielding products, which efficiently prevent the propagation of EMWs. Driven by the urgent need for affordable, durable, miniature, lightweight, and eco-friendly EMI-shielding barriers, various nanomaterials have been rapidly developing [3,4,5].

Common metal-based EMI shielding materials effectively block EMWs; however, challenges regarding their processability, corrosion, and cost limit their wider exploitation [6]. New materials, including conductive polymers, carbon nanotubes, and MXenes, have been utilized as fillers in various polymers, exhibiting excellent shielding effectiveness and other required properties, such as being lightweight, chemically stable, flexible, and processable. These materials reach very high values of total shielding effectiveness at very low thicknesses, such as 84.9 µm thickness and total shielding effectiveness (EMI SE) of 44.56 dB in the X-band for carbon nanotube (CNT)-Fe_3_O_4_ layer (FCFe) and MXene [7], Ti_3_C_2_T_x_ MXene embedded in polyvinyl alcohol yielding 34.80 dB shielding efficiency at 5 µm thickness [8], and Fe_3_O_4_-loaded cellulose/polyacrylonitrile nanofibers with Ti_3_C_2_T_x_ MXene at 640 μm thickness reached EMI SE of 33.2 dB [9]. Considering the ecological burden of plastic materials and the challenges of plastic composite recycling, scientific attention has focused on bio-based materials such as biochar and cellulose scaffolds as potential EMI-shielding materials [10,11,12,13].

Owing to their tunable electrical properties, mechanical strength, light weight, and chemical resistance, graphene and its derivatives have been studied for shielding applications [14,15,16]. When the incident EMWs collide with the graphene surface, they are absorbed, reflected, internally reflected, or transmitted [16]. Dielectric and magnetic losses achieve absorption loss, whereas conduction and polarization losses influence the dielectric loss. Conduction loss is associated with charge migration across graphene sheets and charge hopping from one layer to another, which occurs when materials contain graphene sheets in the construction that are high enough to allow the sheets to create a conductive [17]. This occurs in graphene composites when the mass ratio is above the threshold concentration [17]. This type of wave attenuation depends on the electrical conductivity of the material. In contrast, polarization loss occurs when dipoles in materials under an electrical field are polarized, leading to the attenuation or disappearance of the field [18]. When the dipoles return to their initial state, heat is generated and lost. In graphene-based materials, dipoles stem from polar functional groups, such as O- or N-containing functional groups, owing to the differences in electronegativity between the C atoms in graphene and the heteroatoms. These groups lead to the formation of defect sites where charge carriers can be trapped [19,20]. One strategy to increase EMI shielding effectiveness and electrical conductivity of graphene sheets is to incorporate electron-rich atoms in the graphene structure [21,22]. In the case of n-type doping of graphene, heteroatoms such as N, I, P, B, or S form localized regions with increasing electron density in the π-cloud of graphene sheets, enhancing charge carrier mobility and amplifying polarization at defect sites. It was reported that dipole polarization established between C-N bonds improves the polarization relaxation loss of EMWs, yielding an EMI shielding effectiveness of 45.6 dB in the X-band [22].

Herein, we explored the possibilities of using the thermal treatment of graphene oxides at 2 different temperatures, 500 °C and 800 °C, in a pure NH_3_ atmosphere to achieve N-doping of GO and the effects of treatment on the electrical properties. For the first time, two different starting materials were treated: GO obtained using the modified Hummers’ method, and GO produced using electrochemical exfoliation of highly ordered pyrolytic graphite. Namely, one highly oxidized and disordered Hummers’ GO and the other with fewer oxygen-containing functional groups, and more domains with sp^2^ regions were studied. A similar procedure was previously employed to achieve N-doping of GO [23], where at temperatures above 500 °C, graphene was hydrophobic, with N incorporated in graphene sheets as pyridinic N, pyrrolic-N, and graphitic N, in atomic percentages (at%) between 7.7 and 12.5. Considering that pyridinic and pyrrolic N were identified as factors affecting the polarization relaxation loss, and graphitic N facilitates electron migration and enhances EMW absorption [22], this procedure aimed to improve electrical conductivity and EMI SE of GO. Using different GO as a matrix for N incorporation, we investigated how the precursor affects the ability of N atoms to build N into graphene sheets.

## 2. Results

To investigate the chemical composition and nature of the chemical bonds in the annealed GO, EA, SEM-EDS, TGA, Raman, FTIR, and XRD analyses were conducted.

Table 1 shows the results of the EA, indicating that C is dominantly present in each sample, ranging from 81.04 to 90.00 wt%. The wt% of H was similar in all samples, whereas the wt% of N was the highest for GO-500. Oxygen was detected between 5.69 and 8.58 wt%, while S was identified in GO-500 and HOPG-500 as a residue. The amount of N introduced to the GO samples was inversely proportional to the treatment temperature. In the two sets of samples, the N was incorporated at a higher wt% at 500 °C.

**Figure 1:**
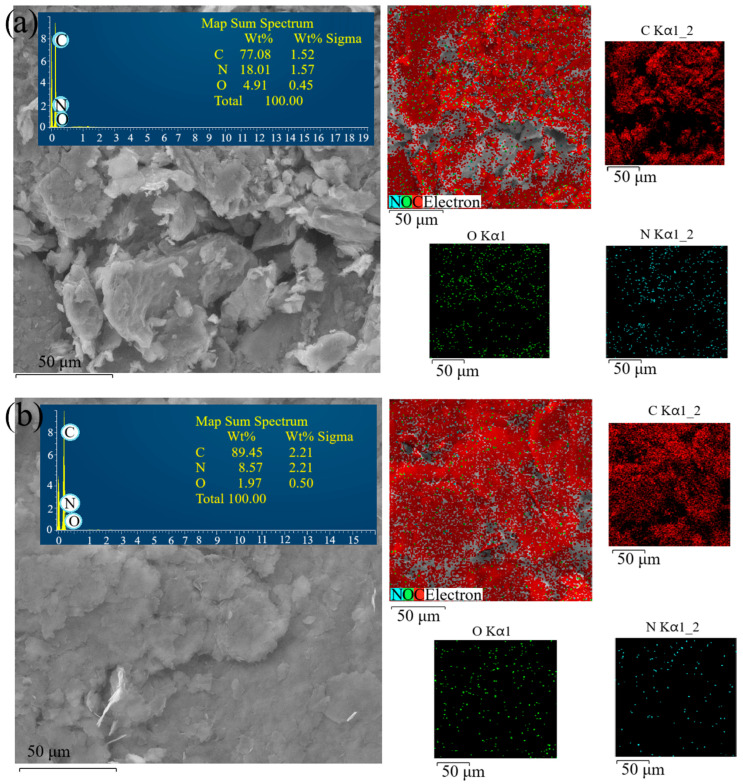
SEM images and EDS maps for GO-500 (**a**) and HOPG-800 (**b**).

The surface chemical composition and morphology of the GO samples were investigated using SEM-EDS analysis. In Figure 1, SEM images and associated EDS maps of the identified elements for GO-500 and HOPG-800 are presented. The SEM images showed layered morphology for both samples, while the element maps indicated a homogeneous and equal distribution of each detected element over the investigated surface. Table S1 (Supporting Information) lists the elemental compositions extracted from the EDS spectra for all the samples, in both wt% and at%. Compared to values in Table 1, EDS analysis showed similar but different wt% values for each element from the elemental analysis. EDS analysis was used to investigate the surface chemical compositions, and the depth of the analyzed specimen depends on the electron beam energy and atomic masses of the constituent elements present in the specimen [24,25]. EA studies the bulk sample that is combusted and analyzed, resulting in values representative of the overall specimen. Thus, similar values are in agreement, considering the difference in the principles of the two analyses and the confirmed results obtained.

**Figure 2:**
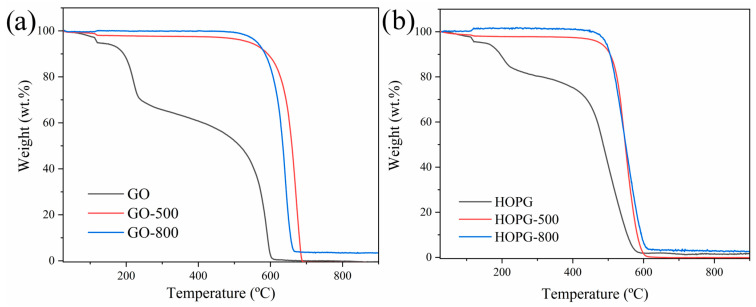
TGA curves of GO, GO-500, and GO-800 (**a**); HOPG, HOPG-500, and HOPG-800 (**b**).

Figure 2 presents the TGA of GO, GO-500, GO-800 (a), and HOPG, HOPG-500, and HOPG-800 (b). The TGA curve of GO shows an initial weight loss attributed to physically bonded water molecules (5.83 wt%) up to a temperature of ca. 150 °C (Figure 2a, black continuous line). This indicates the hydrophilicity of the sample. At ca. 250 °C, a large weight loss was observed (28.58 wt%), which was associated with eliminating oxygen-containing functional groups. The complete combustion of GO occurred at 552 °C. For GO-500, only 1.82 wt% was lost at 150 °C, while for GO-800, the weight loss was 0.01. These results indicate that the polarities of GO-500 and GO-800 were significantly changed, and that both were hydrophobic. At ca. 250 °C, weight loss was not observed, while complete combustion occurred at 608 and 586 °C, GO-500 and GO-800 (red and blue curves in Figure 2a), respectively.

HOPG, HOPG-500, and HOPG-800 samples exhibited similar TGA curves as GO samples (Figure 2b), with temperatures of total combustion of 415, 479, and 481 °C, respectively.

The absence of a weight loss below 200 °C in TG curves suggests that there is no presence of physisorbed water in the samples treated under pure NH_3_ gas at 500 and 800 °C, which is associated with a less hydrophilic character compared with the starting GO. Being a known reducing agent, NH_3_ not only acts as a N source, but also induce the elimination of O-bearing functionalities from the samples, as confirmed by the disappearance of the weight loss associated with these species and the increase in the thermal stability of the material, The thermal stability of the samples closely depends on the concentration of N-containing groups within the conjugated lattice. In agreement with previous reports [23,26], the higher the N content in N-doped rGOs is (500 °C treatments incorporated the larger concentration of N-based groups compared with treatments performed at 800 °C), the higher the thermal stability against oxidation in air was observed.

**Figure 3:**
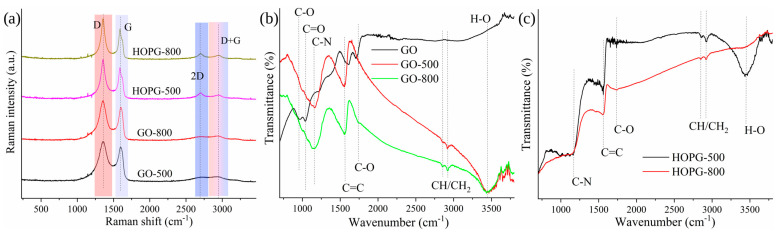
Raman (**a**) and FTIR spectra (**b**) of GO-500, and GO-800; (**c**) HOPG-500, and HOPG-800.

Figure 3 shows Raman (a) and FTIR (b–d) spectra of GO samples annealed under pure NH_3_ gas. All Raman spectra showed the presence of bands at 1355 cm^−1^, assigned to the disordered or as-indicated D-band, at around 1600 cm^−1^, the graphitic or G-band was observed, at 2700 cm^−1^, the so-called 2D band, and the band indicated as D+G (2950 cm^−1^) was also identified [27,28,29]. In the case of HOPG-500 and HOPG-800, G bands are split, due to changes in the bond lengths and angles of graphene sheets as a result of strain [30]. Namely, external perturbations of the hexagonal symmetry of graphene occurred due to the introduction of N-functional groups in the graphene sheets. In all spectra, the D band’s intensity is higher than the G bands, indicating high disorder in the graphitic structure of the samples. The calculated values of the intensity ratios between D and G bands are listed in Table 2.

Table 2 shows that the highest structural order was calculated for GO, while the most disordered sample is HOPG-800, according to I_D_/I_G_ values. Additionally, the position of the G band is associated with the graphitic regions in graphene-based materials [30]. Using the Knight and White equation [31], the in-plane crystallite size (La) was estimated and listed in Table 2. The values were calculated using the relation La = 4.4 (I_D_/I_G_)^−1^, and indicate the lowest crystalline size for sample HOPG-800. Compared to other N-doped graphene, where La was 16.5 nm [32], our samples showed significantly lower in-plane crystallite size, while I_D_/I_G_ values are similar. Furthermore, the positions of 2D (2699 cm^−1^) in HOPG-500 and HOPG-800 (2702 cm^−1^) are similar to the previously reported N-doped GO [32].

FTIR spectra were used to investigate the binding nature of the elements identified using SEM-EDS and EA. In case of GO, vibrations associated with H-O (ν~3200–3400 cm^−1^) from physically absorbed water or OH groups, C=O (ν~1700 cm^−1^) from carboxyl, C=C (ν~1590 cm^−1^) from aromatic domains, O–H (β(OH)) (ν~1372 cm^−1^), C=O (ν~1223 cm^−1^), C–O in C-O-C (ν~1042 cm^−1^), and C-O in epoxy (ν~ 952 cm^−1^) were observed [33,34]. In FTIR spectra of GO-500 and GO-800, the band at 3400 cm^−1^ significantly increased. Moreover, two new bands at 2922 and 2853 cm^−1^ from CH/CH_2_ were detected. The band at 1700 cm^−1^ vanished in the GO-500 spectrum, while in the case of GO-800, a lower intensity and shifting up to 1739 cm^−1,^ owing to the complete or partial removal of carboxyl groups, occurs. Finally, the 1590 cm^−1^ band shifted to 1552 cm^−1^ in GO-500 and GO-800 FTIR spectra. Both spectra show bands at 1160 cm^−1^, which were associated with C-N vibrations [35,36], while bands at 1042 and 952 cm^−1^ almost completely vanished, indicating the removal of epoxy groups.

In the case of HOPG, HOPG-500 and HOPG-800 samples showed strong bands attributed to C-N (ν~1161 cm^−1^), C=C (ν~1551 cm^−1^) from aromatic domains, C=O (ν~1737 cm^−1^), and CH/CH_2_ (2922 and 2853 cm^−1^) vibrations. A strong band at 3400 cm^−1^ was detected in the HOPG-500 sample, while for the HOPG-800 sample, this band was not detected. The reason for this change is associated with an alteration in the polarity of HOPG-800 and an increase in its hydrophobicity, resulting in a lowering of the tendency of the material to absorb atmospheric water physically.

All samples showed evident changes in FTIR spectra after thermal treatment under pure ammonia gas. At high temperature, ammonolysis led to significant changes in functional groups anchored to the graphene oxide. The new bands assigned to C-N bonds and detected in all thermally modified samples correspond to N incorporated into the graphitic lattice. Moreover, the simultaneous elimination of the O-containing moieties from the GO was confirmed by the disappearance of the bands corresponding to hydroxyl (1042 cm^−1^) and epoxy groups (952 cm^−1^).

**Figure 4:**
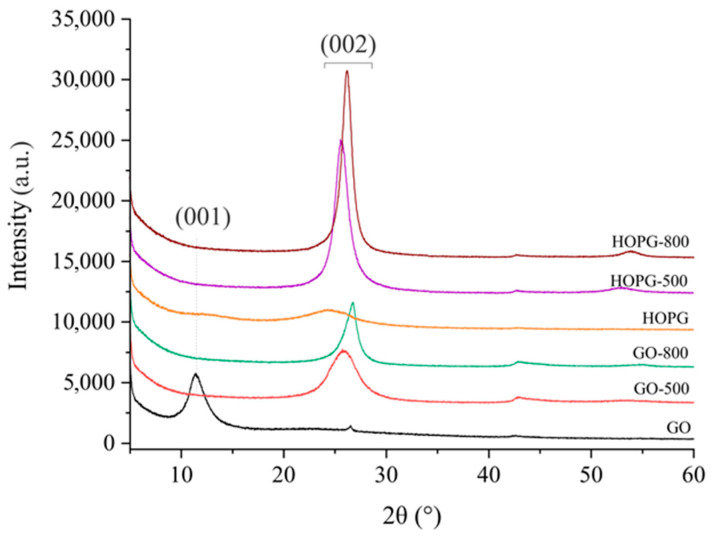
XR diffractograms of GO and HOPG, and thermally treated GO and HOPG samples.

The crystal structure of the samples was analyzed by X-ray diffraction (Figure 4). The XRD profile of the initial GO has the (001) reflection at 2θ = 11.4°, corresponding to an interplanar distance of approximately 7.8 Å [37,38]. On the other hand, pristine HOPG shows two broad peaks, at 2θ ≈ 24.4 ° and 2θ = 12.4°, which correspond to the (002) and (001) reflections, respectively [39]. The deviation from the literature value for the (002) reflection of exfoliated graphene (2θ = 26°) and its broadness might be the consequence of the corrugated graphene’s structure and the increased interlayer spacing [40]. Thermally treated GO samples showed an emergence of a new feature (002) at 2θ ≈ 25° and a simultaneous disappearance of the (001) reflection. The (002) feature of GO and HOPG samples thermally treated at 800 °C was shifted towards a higher angle than samples treated at 500 °C. This implies a slight change in the interplanar distances toward lower values for the samples treated at the higher temperature, owing to the elimination of hydroxyl and epoxy groups from graphene’s surface under heating, as observed previously in FTIR spectra (Figure 3b,c).

**Figure 5:**
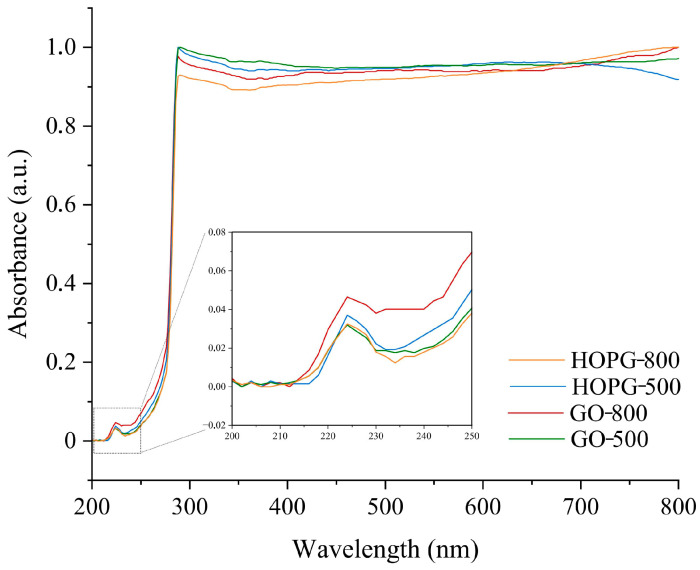
UV-VIS spectra of GO-500, GO-800, HOPG-500, and HOPG-800.

The UV-Vis absorption spectra of thermally treated GO and HOPG were recorded in toluene and presented in Figure 5. All spectra show an absorption peak at 224 nm, corresponding to the π-π* transitions of aromatic C-C bonds [41,42]. The peak was not shifted in position after the annealing at 800 °C. Interestingly, the spectrum of GO showed an additional feature at 255 nm. This new band could be associated with N-functional groups [43].

Further investigations of the optical and electrical properties of GO, HOPG, and N-doped samples were carried out by recording reflection spectra. The Tauc equation was used to calculate the optical band gap. Figure S1 shows the corresponding Tauc plots, (αhν)^2^ as a function of hν, where *α* is the absorption coefficient, *h* is the Planck constant, and *ν* is the frequency [44,45]. The value of the optical energy band gap (Eg) was estimated as the x-intercept of an extrapolated Tauc plot, and the obtained values are listed in Table 3. It was observed that the Eg was decreased significantly after thermal treatment, from 4.88 eV to 1.88 eV. In the case of N-doping of GO produced by electrochemical exfoliation of HOPG, Eg was lower, and a small decrease was measured after thermal treatment at 500 °C and 800 °C, with the lowest value calculated for the HOPG derivatives corresponding to the sample treated at 500 °C. The lowering in the values of Eg was previously reported for chemically reduced GO [45,46] or GO reduced by specific drying conditions [47]. A decrease in the Eg values leads to extended absorption in the visible part of the electromagnetic spectrum [41], improved semiconductor properties [46,48], and is associated with the restoring π-domains in graphene sheets. These results are in agreement with XRD and FTIR analyses, where removal of O-functional groups and improvement of sp^2^ domains were reported.

**Figure 6:**
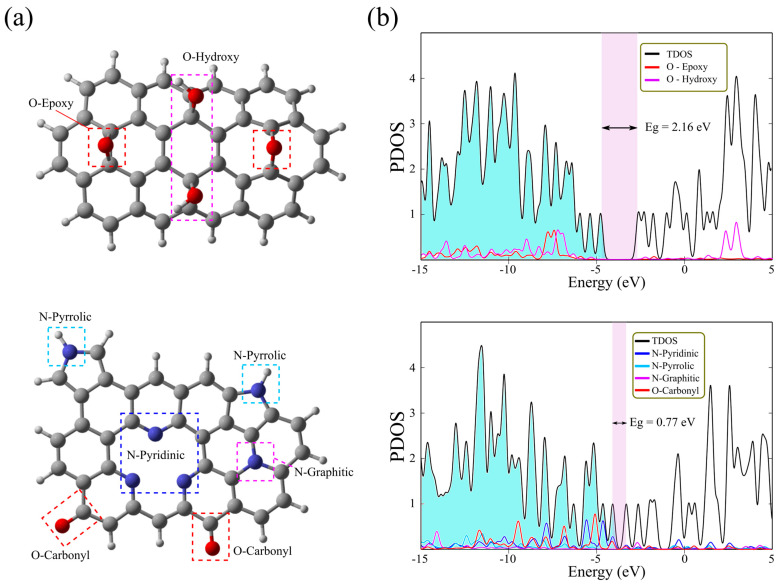
(**a**) The optimized structures of [C_40_H_16_O_2_(OH)_2_] and [C_35_H_14_O_2_N_6_] clusters, (**b**) the total and partial density of states (TDOS/PDOS) diagrams.

We used DFT calculations to investigate the electronic structure of N-doped rGO and compare it to that of rGO. Ab initio methods can provide an atomistic model of variously functionalized and N-doped rGO, revealing their optical and electronic properties. We employed these techniques to calculate the total and partial density of states (TDOS/PDOS) diagrams of rGO and N-doped rGO. These results provided information about the energy structure changes mainly occurring around the Fermi level when nitrogen atoms are embedded into the graphene core. Figure 6 shows the optimized structures of [C_40_H_16_O_2_(OH)_2_] and [C_35_H_14_O_2_N_6_] clusters with the corresponding density of states (TDOS/PDOS) diagrams. The [C_40_H_16_O_2_(OH)_2_] cluster, with two basal epoxy and hydroxyl groups, is constructed to mimic rGO, while the [C_35_H_14_O_2_N_6_] cluster represents N-doped rGO. The [C_35_H_14_O_2_N_6_] cluster has one graphitic, two pyrrolic, and three pyridinic nitrogen atoms embedded into the graphene lattice. The density of states diagram of the cluster representing rGO indicates that the bandgap is 2.16 eV, which categorizes it as a semiconducting material (Figure 6b). This value is close to the experimentally determined bandgap of reduced GO. The energy states of two types of oxygen atoms (from epoxy and hydroxyl groups) are almost overlapping in the valence region and do not take part in the states near the Fermi level. On the other hand, the cluster corresponding to N-doped rGO has a reduced bandgap of 0.77 eV, indicating increased electrical conductivity compared to rGO, as demonstrated experimentally (Figure 6b). The PDOS diagram shows that the electronic levels of pyridinic nitrogen atoms are near the Fermi level, while the pyrrolic and graphitic nitrogen levels are located deep in the valence band. The electronic states of carbonyl oxygen atoms also appear as part of the frontier molecular orbitals and extend across the entire valence region.

The influence of the structure of staring GO has been investigated for the first time. Previous papers have investigated the effects of different structures of N sources and explored their efficiency to incorporate N in a graphene sheet [49,50,51], finally reporting that N-precursors affected the at% of incorporated N and the N-functional groups.

It is well known that the dissociation of ammonia into N_2_ and H_2_ begins appreciably at temperatures above 500 °C; however, this process is relatively slow and closely depends on other aspects such as gas flow rates and reaction times [52]. Here, we have employed high flow rates (300 mL/min) to significantly minimize the NH_3_ decomposition and improve the doping process. As a consequence, the reduction of the system was mostly attributed to the effect of NH_3_ molecules, instead of H_2_ that may incipiently be generated during the treatment. Increasing the temperature of treatment up to 800 °C favors the dissociation of NH_3_ into H_2_ and N_2_. As a consequence, fewer NH_3_ molecules interact with the reactive O-containing sp^3^ groups, thus leading to a sample with a lower N content. In this sample, however, the highest degree of reduction is attributed to thermal decomposition of labile species attached to the GO surface.

Herein, we employed thermal treatment of the different graphene oxides at 500 °C and 800 °C, under pure NH_3_ atmosphere. The first one was produced using the modified Hummers method, while the additional GO was produced by electrochemical exfoliation of HOPG. The ability of N from NH_3_ to be incorporated into different GOs was tested. After the treatment under selected conditions, all GO had a lower O content, improved thermal stability, and N incorporated in the graphene crystalline structure. Between two different starting materials, significant differences in N-doping were measured. At 500 °C, GO showed one of the highest reported N-doping in the literature (11.25 ± 0.08 at%), while HOPG had only 5.85 ± 0.03 at% of N in its structure. A similar trend was observed in samples treated at 800 °C, but the at% resulted in being lower than at 500 °C. It was previously discussed that N species react with most labile sites in the GO lattice, O atoms on the defects and vacancy sites, and/or replace the C atoms [53].

NH_3_ molecules reacted mainly with COOH groups, creating amide GO, imide GO, and lactam GO, which turned into pyridinic and pyrrolic groups in GO sheets, after tautomeric isomerization [49]. The highest N-doping of Hummers GO could be explained by the largest number of defects, vacancies with labile bonds, as a potential place for N-bonding. The GO from HOPG showed the lowest O content, and consequently, the lowest number of potential N-doping sites. Thus, the lowest N-content was measured in the HOPG-500 sample.

To investigate the effects of the structural modification on the electrical properties, a 4-point probe was used to measure the sheet resistance, R_s_, and the calculation of electrical conductivity, σ. These results are listed in Table 4. The obtained values of R_s_ are similar to sheet resistance measured in other carbon-based nanomaterials and their composites [54,55]. GO from the non-conductive material became more conductive after being annealed at 500 °C and increased with temperature. In the case of HOPG, R_s_ and σ showed that the material is electrically conductive, while treatment at 500 and 800 °C further improved electrical conductivity.

**Figure 7:**
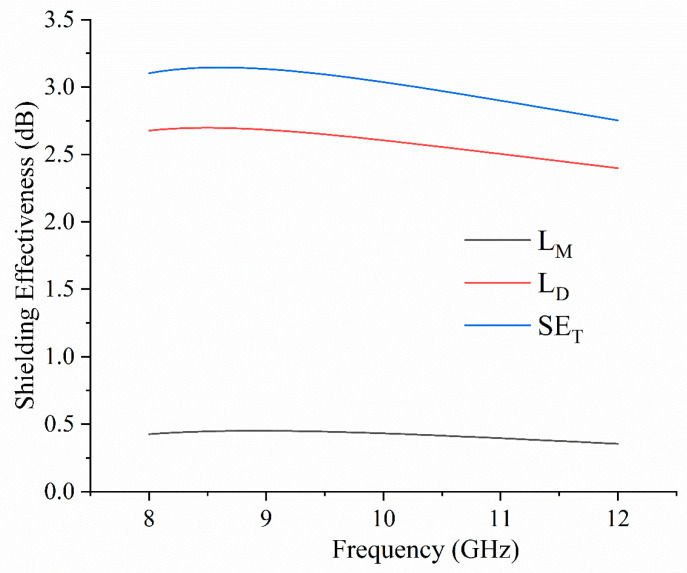
SE_T_, L_M_, and L_D_ values for GO-500, measured in the 8–12 GHz frequency range.

The total effectiveness (EMI SE) of N-doped GO to block the propagation of electromagnetic waves in the frequency range between 8 and 12 GHz was investigated. A powdered GO-500 sample was prepared, and the microstrip approach was used. In Figure 7, the shielding effectiveness of GO-500 is presented. The total shielding effectiveness (SE_T_) was estimated using Equations (1)–(3) [56,57]:

SE_T_ = L_D_ + L_M_, (1)

where SE is obtained as a sum of dissipation (L_D_) and mismatch (L_M_) loss. The L_M_ was obtained using Equation (2):

L_M_ = −10log_10_(1 − |S_11_|^2^), (2)

and the value of L_D_ was estimated using the S_11_ (reflection scattering parameter), and S_21_ (transmission scattering parameter), according to Equation (3):

L_D_ = −10log_10_((|S_21_|^2^)/(1 − |S_11_|^2^). (3)

The values of scattering parameters S_11_ and S_21_ were recorded using a VNA.

The sample shows limited ability to block EMWs in the 8–12 GHz frequency range. We measured only 3.2 dB at a frequency of 8.5 GHz. Compared to GO [58], which was completely transparent to incident EMWs in the 8–12 GHz frequency range, this result indicated that N-doped GO is a more efficient shielding material.

In total SE, the main contribution to the shielding is attributed to the dissipation loss. The EMI SE associated with the mismatch loss showed limited contribution to the total EMI SE, considering that mismatch loss is associated with wave reflection. At the same time, dissipation is a result of EMW absorption. The presented results indicate that the main shielding mechanism of EMW attenuation for GO-500 is absorption. A similar mechanism was previously reported in biochar [59]. Thermal treatment of GO contributed to EMI shielding in several ways:

- Considering electronic levels of pyridinic N are near the Fermi level, while the pyrrolic and graphitic N levels are deeply located in the valence band, the electron density is higher;
- The electrical energy band gap is lowered, making the material more electrically conductive;
- Doped GO and HOPG showed drastically lower sheet resistance;
- The sp^2^ region of all thermally treated samples was increased;
- Formation of C-N bonds improved the polarization loss.

Although the measured shielding effectiveness is lower than values reported in the literature for N-doped GO (45.6 dB [22], 30 dB [21], 35 dB [60]), the results collected in this study show the method’s promising potential to convert GO without any shielding effects [58] into materials with a shielding effectiveness of 3.2 dB.

## 3. Materials and Methods

### 3.1. Materials

GO was synthesized using a modified Hummers method [61]. First, 1 g of graphite pellet (type Z-346 KS6, TIMREX^®^, Bodio, Switzerland) was sonicated in sulfuric acid (23.3 mL, 96%, Carlo Erba, Vigevano, Italy) in an ice bath. Then, the reaction mixture was transferred to a mechanical stirrer, and 3 g of KMnO_4_ was gradually added under vigorous stirring for 30 min. Subsequently, the temperature was gently increased to 40 °C, and 50 mL of demineralized water was slowly added. The temperature was maintained at 40 °C for 30 min, and then increased to 90 °C for 15 min. Afterwards, the reaction was cooled to room temperature and stopped by pouring the reaction mixture into water (500 mL) with H_2_O_2_ (14.7 mL of 30 vol%, Carl Roth, Karlsruhe, Germany). GO was cleaned in several steps, as previously described [61]. Purified GO was used for thermal modification and was named GO.

The HOPG sample was produced by electrochemical exfoliation of highly oriented pyrolyzed graphite (HOPG) [62,63]. HOPG was previously produced at the Vinca Institute of Nuclear Sciences, Vinca, Serbia, and used as a cathode and anode in an electrochemical cell. The distance between electrodes was set at 4 cm. An electrolyte, (NH_4_)_2_S_2_O_8_ (99.99%, Centrohem, Belgrade, Serbia), was dissolved in water (0.1 M) was used. The voltage applied to the electrodes was set to +12 V for several hours. The reaction was stopped when the electrodes were oxidized, and a black precipitate was formed at the bottom of the electrochemical cell. The electrochemically oxidized material was sonicated for 1 h in a sonication bath to achieve graphene exfoliation. Centrifugation (3500 rpm for 30 min) was then applied to remove heavy graphitic particles. The supernatant was collected and cleaned of the electrolyte by dialysis using a Spectra/Por (Carl Roth, Karlsruhe, Germany) dialysis membrane with a molecular weight cutoff (MWCO) of 3500 Da. The sample was cleaned for 5 days, after which the dispersion from the bags was collected and dried under reduced pressure.

N-doped reduced graphene derivatives were prepared by annealing powdered GO and HOPG samples (100–300 mg) at both 500 and 800 °C, for 60 min in the presence of a continuous flow (300 mL min^−1^) of pure NH_3_ gas (99.99%, Carburos Metálicos, Barcelona, Spain). For the treatment, each sample was spread on an Al_2_O_3_ boat and placed inside a silica tube at the center of a sandwich-like furnace (Gallur, Zaragoza, Spain). The samples were named GO-500, GO-800, HOPG-500, and HOPG-800, depending on the starting materials and annealing temperatures.

### 3.2. Methods

First, elemental analysis (EA) was used to evaluate the chemical compositions of GO, HOPG, and N-doped samples. We used an AZ Element microanalyzer (model Flash 2000) and an A8 Element microanalyzer (model Flash Smart) (Thermo Fisher Scientific, Waltham, MA, USA). Measurements were carried out using the modified Pregl-Dumas (dynamic flash combustion) method with helium as a flowing gas. The results were registered as weight percentages for each element, resulting from a comparison with the values of a known standard, following the k-factors method.

A high-resolution scanning electron/focused ion beam (dual-beam) Tescan^®^ LYRA 3 FEG/XMH (Brno, Czech Republic) scanning electron microscope (SEM) was used to examine the GO samples. Double-sided carbon tape was used to fix the samples to the supports. SEM images were obtained in a high vacuum at an acceleration voltage of 10 kV. The chemical composition was investigated using a SEM-EDS INCAx-act LN2-free analytical silicon drift detector of characteristic X-rays with PentaFET^®^ Precision (Oxford Instruments, Oxfordshire, UK) with a Tescan^®^ Mira3 XMU, SE detector.

Thermogravimetric analysis (TGA) was performed using a Netzsch instrument (model STA 449 F1 Jupiter^®^, Selb, Germany). Measurements were conducted in air at a heating rate of 10 °C/min. Approximately 3 mg of each sample was used for TGA, and the measurements were repeated twice for each sample.

Raman spectra were obtained with a DXR Raman microscope (Thermo Fisher Scientific, Waltham, MA, USA). A laser wavelength of 532 nm and a power of 2 mW was used. The acquisition time was 10 × 10 s. Samples were analyzed at three different locations, and average spectra were used for analyses. FTIR spectra were collected using a Nicolet iS20 spectrometer (Thermo Fisher Scientific, Waltham, MA, USA). FTIR spectra were recorded using the KBr pellet technique. Pellets were prepared by thoroughly grinding the sample with spectroscopic-grade KBr at a constant ratio of 1 mg of sample to 150 mg of KBr, followed by pressing into transparent disks. All spectra were collected under identical conditions. X-ray diffraction (XRD) analysis was performed using a Philips PW 1050 powder diffractometer (Philips, Amsterdam, The Netherlands) equipped with Ni-filtered Cu Kα radiation and configured in the Bragg–Brentano geometry. Data were collected over a 2θ range of 5–60°, with a step increment of 0.02° and a counting duration of 5 s per step. The interlayer distances were calculated by applying Bragg’s law, considering the wavelength of the incident X-rays of 1.542 Å.

The optical properties were investigated using absorption and reflection spectroscopy. UV-Vis spectra were recorded in the range of 190–800 nm, with a resolution of 2 nm, using an LLG-uniSPEC 2 spectrophotometer (Lab Logistic group, Mekenheim, Germany). GO-500, GO-800, GO-HOPG-500, and GO-HOPG-800 were dispersed using an ultrasonic bath for 30 min in toluene at a 0.03125 mg mL^−1^ concentration. Furthermore, the diffusion reflection of the graphene-based samples was measured using a UV-2600 spectrophotometer, series 2600i (A12596001168) (Shimadzu Scientific Instruments, Inc., Columbia, MD, USA). The reflection spectra were recorded in the 200–1400 nm wavelength range, at medium speed, and at room temperature. Powdered samples were placed between two quartz glasses, and the reflectance spectra were collected.

Gaussian 09, Revision B.01 software was used for all computations [64]. We used density functional theory (DFT) to investigate the electronic structures of rGO and N-doped rGO. To this end, we constructed two clusters with the following gross formulas: [C_40_H_16_O_2_(OH)_2_] and [C_35_H_14_O_2_N_6_]. DFT calculations were performed using the B3LYP-D3 functional [65,66,67] with Pople’s double-ζ polarized 6–31G(d,p) basis set [68]. The structures were fully optimized in the gas phase with subsequent frequency calculations, confirming that they were true minima with no imaginary frequencies. The molecular representation of the clusters was obtained using GaussView 5.0.9 software, and total and partial density of states (TDOS/PDOS) diagrams were obtained using GaussSum 3.0.

For the sheet resistance evaluation, 4-point measurements were performed. Powdered samples of GO, HOPG, GO-500, GO-800, HOPG-500, and HOPG-800 were dispersed in a mixture of ethanol (99%, HPLC purity, J.T. Baker, Phillipsburg, NJ, USA) and demineralized water in a volume ratio of 1:1, in a concentration of 1 mg/mL, using an ultrasonic bath, for 30 min. The stable dispersions were deposited on a membrane filter (IsoporeTM membrane filter, pore size 0.22 μm, GTTP02500, polycarbonate, Merk, Darmstadt, Germany) using a vacuum filtration system. The inner diameter of the vacuum funnel was 1.45 cm, and 14.59 mL of dispersion was deposited. The sheet resistance was measured directly on the membrane using a solid sample. The sheet resistance was measured with a 4-point probe Jandel RM3000+ (Jandel Engineering, Leighton Buzzard, UK) test unit. The distance between the probes was 1 mm. The sheet resistance was measured at five sites on each sample, and the average values were calculated.

To investigate the shielding effectiveness, a microwave experimental setup was used. A Vector Network Analyzer (VNA, compact Streamline 5008A, Keysight Technologies^®^, Santa Rosa, CA, USA) was used to measure the amplitude of the transmission coefficient (S_21_) in the 8 GHz to 12 GHz frequency range.

## 4. Conclusions

The thermal treatment of GO under NH_3_ atmosphere was employed to N-dope and reduce GO in one synthetic step. It was observed that not only the temperature but also the structure of the starting GO affects the at% of the incorporated N. The highest N-doping was measured in Hummers’ GO, and the lowest in GO produced by electrochemical exfoliation of graphite. All N-doped GO showed improved thermal stability, with complete combustion above 500 °C. Although N-doped samples showed a high structural disorder, the enlargement of the sp^2^ domains is evident by XRD analysis. Selected treatment drastically altered the electrical properties of GO, from non-conductive (Rs of ∞) to conductive (Rs = 43.6 ± 3.1 Ω/□). These changes were explained by lowering the electrical band gap, estimated to be 0.74 eV by DFT. The proposed method offers a unique approach to improve the electrical properties of graphene oxide in an eco-friendly, time-saving approach transferable to a scale level and possibility to tune structural, thermal, optical, EMI shielding, and electrical properties of graphene oxide by changing the conditions and starting materials.

## Figures and Tables

**Table 1:** Elemental compositions of samples in wt% measured using an element microanalyzer.

Sample	C ± STD	H ± STD	N ± STD	S ± STD	O ^1^
**GO-500**	81.04 ± 0.21	0.69 ± 0.03	11.25 ± 0.08	0.21 ± 0.01	6.81
**GO-800**	83.63 ± 0.19	0.68 ± 0.05	7.11 ± 0.06	<0.1	8.58
**HOPG-500**	87.93 ± 0.08	0.34 ± 0.01	5.85 ± 0.03	0.19 ± 0.02	5.69
**HOPG-800**	90 ± 0.13	0.41 ± 0.01	3.46 ± 0.02	<0.1	6.13

^1^ The values of O wt% were calculated by subtracting the wt% of the measured elements (C, H, N, and S).

**Table 2:** Position of G bands, I_D_/I_G_ ratios, and La values.

Sample	G Band (cm^−1^)	I_D_/I_G_	La
**GO**	1596	1.11	4.35
**GO-500**	1603	1.15	3.82
**GO-800**	1602	1.20	3.70
**HOPG-500**	1587	1.25	3.55
**HOPG-800**	1589	1.33	3.34

**Table 3:** Optical band gap of GO and HOPG powders.

Sample	E_g_ (eV)
**GO**	4.88
**GO-500**	1.88
**GO-800**	1.85
**HOPG**	1.95
**HOPG-500**	1.82
**HOPG-800**	1.83

**Table 4:** Sheet resistance and electrical conductivity of GO and HOPG thin films at 1 mA.

Sample	R_s_ (Ω/□)	σ (S/m)
**GO**	∞	~0
**GO-500**	196.0 ± 1	283.4
**GO-800**	224.0 ± 3	248.0
**HOPG**	45.0 ± 2.7	1234.6
**HOPG-500**	7.4 ± 0.6	7507.5
**HOPG-800**	6.7 ± 0.4	8341.67

## Data Availability

Datasets analyzed in the current study are available in the Zenodo repository (https://doi.org/10.5281/zenodo.16164681).

## Supplementary Materials

The following supporting information can be downloaded at https://www.mdpi.com/article/10.3390/molecules30173579/s1; associated data are available at https://doi.org/10.5281/zenodo.16164681. Table S1. Elemental composition in wt% and at% for all samples. Figure S1. Tauc plot of GO (a), GO-500 (b), GO-800 (c), HOPG (d), HOPG-500 (e), and HOPG-800 (f).

## Abbreviations

The following abbreviations are used in this manuscript:

GO	Graphene oxide
HOPG	Highly Ordered Pyrolytic Graphite
EMWs	Electromagnetic Waves
EMI	Electromagnetic Interference
SEM	Scanning Electron Microscope
TGA	Thermogravimetric analysis
DFT	Density Functional Theory
FTIR	Fourier Transform Infrared Spectroscopy
XRD	X-Ray Diffraction
VNA	Vector Network Analyzer
