# Supplementary material for: A New Route to Tune the Electrical Properties of Graphene Oxide: A Simultaneous, One-Step N-Doping and Reduction as a Tool for Its Structural Transformation
Source: Molecules. 2025 Sep 1;30(17):3579. doi: 10.3390/molecules30173579 (PMC12429991; doi:10.3390/molecules30173579)
Supplement: Supplementary file 1 [file molecules-30-03579-s001.zip › molecules-3840233-supplementary.pdf]

Supporting Information

Article

A New Route to Tune the Electrical Properties of Graphene Oxide: A Simultaneous, One-Step N-Doping and Reduction as a Tool for Its Structural Transformation

Andjela Stefanović 1, Muhammad Yasir 2,*, Gerard Tobías-Rossell 3, Stefania Sandoval Rojano 3, Dušan Sredojević 1, Dejan Kepić 1, Duška Kleut 1, Warda Saeed 2, Miloš Milović 1, Danica Bajuk-Bogdanović 4 and Svetlana Jovanović 1,*

1 Vinča Institute of Nuclear Sciences-National Institute of the Republic of Serbia, University of Belgrade, P.O. Box 522, 11000 Belgrade, Serbia
2 Carl von Ossietzky Universität Oldenburg, 26129 Oldenburg, Germany
3 Institut de Ciencia de Materials de Barcelona (ICMAB-CSIC), Campus de la UAB, 08193 Bellaterra, Barcelona, Spain; gerard.tobias@icmab.es (G.T.-R.); ssandoval@icmab.es (S.S.R.)
4 University of Belgrade, Faculty of Physical Chemistry, Studentski trg 12-16, 11158 Belgrade, Serbia; danabb@ffh.bg.ac.rs
* Correspondence: muhammad.yasir@uni-oldenburg.de (M.Y.); svetlanajovanovic@vin.bg.ac.rs (S.J.)

Table S1. Elemental composition in wt% and at% for all samples.

Sample	C in wt%/at%	N in wt%/at%	O in wt%/at%	S
**GO**	54.26±0.52/61.89	0	43.63±0.51/37.36	1.15±0.04/0.49
**GO-500**	77.08±1.52/80.12	18.01±1.57/16.05	4.91±0.45/3.83	0
**GO-800**	84.05±1.42/86.28	12.96±1.44/11.41	2.99±0.35/2.30	0
**HOPG-500**	84.71±1.72/87.03	10.78±1.76/9.49	4.51±0.45/3.48	0
**HOPG-800**	89.45±2.21/91.01	8.57± 2.21/7.48	1.97±0.50/1.51	0

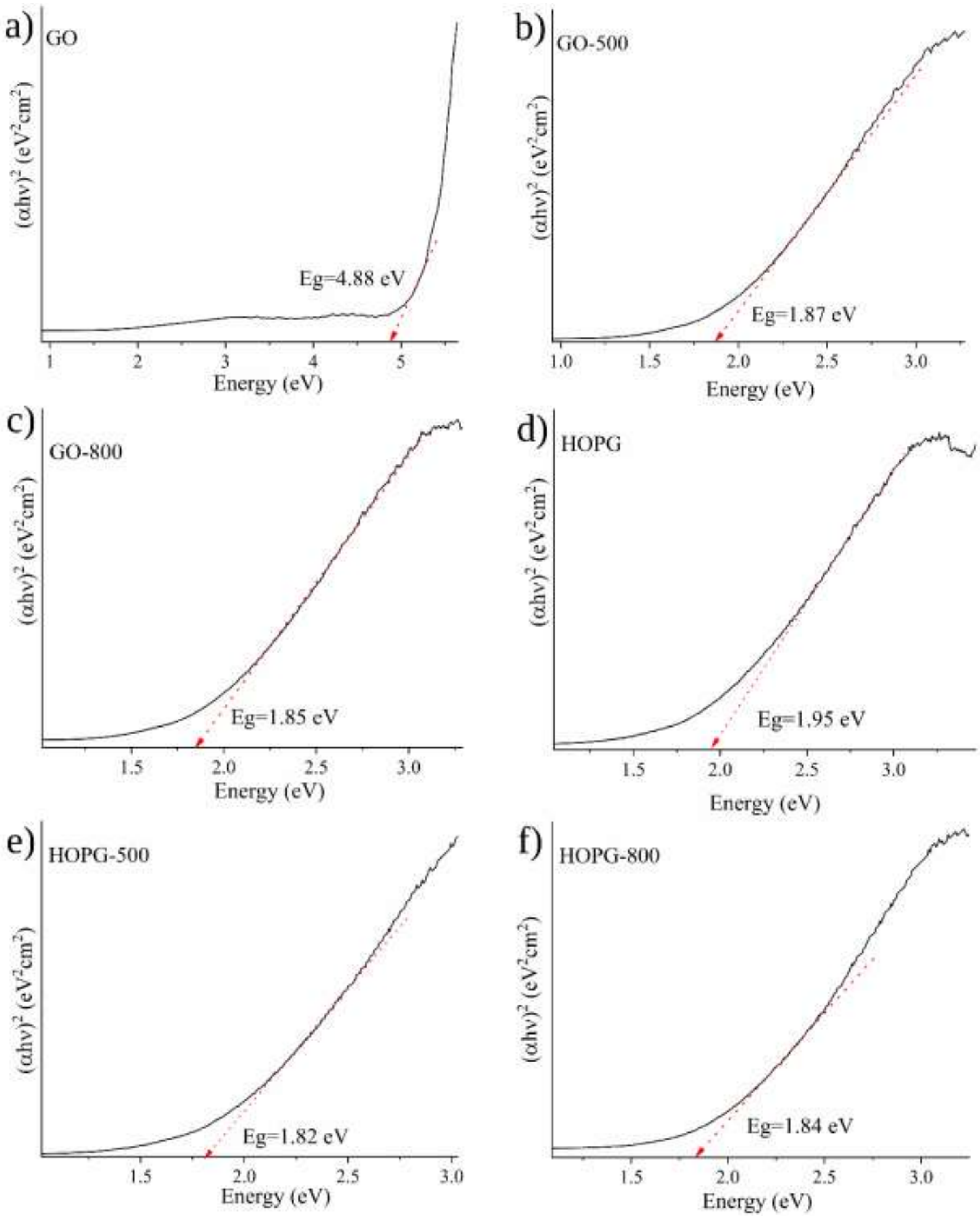

Figure S1. Tauc plot of GO (a), GO-500 (b), GO-800 (c), HOPG (d), HOPG-500 (e), and HOPG-800 (f).
